# Anatomical basis for the development of a thoracic duct cannulation model without thoracotomy in Large White pigs

**DOI:** 10.1186/s12917-015-0430-9

**Published:** 2015-05-14

**Authors:** Hung-Hsun Yen, Christina M Murray, Helen MS Davies

**Affiliations:** Faculty of Veterinary and Agriculture Science, The University of Melbourne, Parkville, VIC 3010 Australia

**Keywords:** Thoracic duct, Anatomy, Swine model

## Abstract

**Background:**

To collect lymph draining the lungs provides a useful strategy for tracing pulmonary microvascular fluid and protein biology. A methodology that allows for *in vivo* sampling of efferent pulmonary lymph in real-time in sheep by cannulating the thoracic duct without entering the thoracic cavity was previously established. To develop a similar thoracic duct cannulation model without thoracotomy in pigs, we investigated the anatomy of the left cervico-thoracic regions of 15 Large White (Yorkshire or Yorkshire-dominated) piglets (aged 4–7 weeks).

**Results:**

The thoracic duct, together with the left tracheal trunk, joined the cardiovascular system (the ampulla of the thoracic duct) at a site located craniomedial to the first rib on the left in 80 % (12/15) of the piglets.

**Conclusions:**

As the location of the ampulla of the thoracic duct was consistent in most of the piglets, Large White piglets appear to be suitable for the development of a thoracic duct cannulation model without thoracotomy. The anatomical findings in this study will enable the development of further surgical procedures for cannulating the thoracic duct without thoracotomy, with minimal damage to local tissue, and without transecting any major blood vessels, nerves or muscle bellies. The establishment of a thoracic duct cannulation model for collecting *in vivo*, *in situ* efferent lymph, including pulmonary lymph, in pigs without entering the thoracic cavity would be invaluable for many immunological studies, studies on pulmonary immune responses in particular.

## Background

The successful cannulation of the terminal part of the thoracic duct in the left pre-scapular region in sheep was initially described by Kassai et al. in 1972 (in Hungarian) [1]. In that study, they used the model to study *Dictyocaulus filaria* infection. In 2009, Yen et al. reported another successful preparation of a pulmonary lymph fistula through thoracic duct cannulation without thoracotomy using surgical approaches different to that of Kassai et al. [2]. These models provided a strategy for monitoring *in vivo*, *in situ* pulmonary pathology and immunobiology in real-time in sheep. It would be of great benefit to pulmonary studies if the model for *in vivo* real-time pulmonary lymph collection could be established in pigs. However, there are few references for pigs that describe the anatomy of the thoracic duct at the point where it joins the cardiovascular system (the ampulla of the thoracic duct), its relationship to other anatomical structures and the anatomical variations within the same pig breed [3]. Further, photographic images showing genuine fresh tissues of the thoracic duct and associated anatomical structures in the pig are not available in the literature.

In pigs, pulmonary afferent lymph from the lungs drains into the tracheobronchial and cranial mediastinal lymph centres and then enters the thoracic duct [4]. Consequently, harvesting lymph from the thoracic duct provides a strategy to sample lymph that contains lymph draining the lungs and the local lymph centres. Changes to the composition of thoracic duct lymph may indicate functional processes occurring in the pulmonary system. However, thoracic duct lymph contains lymph draining several other body systems. Hence, changes in thoracic duct lymph composition may also result from the effects of processes in other body systems. When the lungs are the key target organs of pathogens or the corresponding treatments i.e. intra-bronchial delivery of treatments using a bronchoscope, changes in the composition of thoracic duct lymph should primarily come from the pulmonary lymph rather than from the other tissues.

In this study, we investigated the anatomy of the thoracic duct where it joins the cardiovascular system, and its relationship to the other major anatomical structures at the thoracic inlet in Large White piglets (also known as the Yorkshire or Yorkshire-dominated piglets) - a key pig breed usually accessible in the pig industry in Victoria, Australia and a number of other countries in the world. We believe that the results will be of help in identifying the lymphatic vessels and their relationships to the other structures at the thoracic inlet on the left side in pigs. These results provide the basis for the development of a pig model in which the thoracic duct and the tracheal trunk (left) are cannulated without thoracotomy for respiratory studies.

## Results

**Fig. 1:**
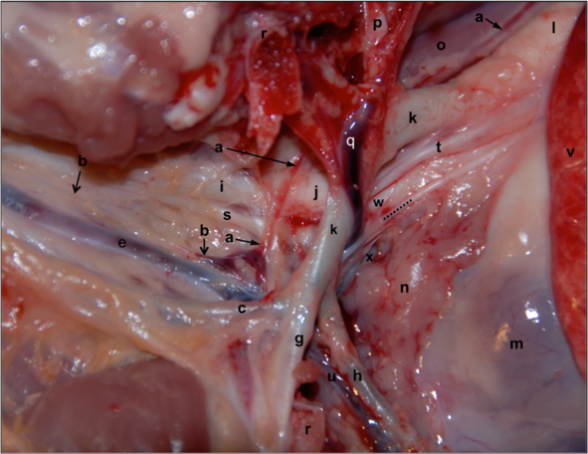
The course of the thoracic duct and the tracheal trunk in the cervicothoracic region on the left. Dissections were performed on a 6-week old Large White pig cadaver to expose the craniolateral aspect of the left cervicothoracic region. The left forelimb was retracted craniolaterally. The tracheal trunk anastomosed with the thoracic duct (the ampulla of the thoracic duct) in this pig. The picture is positioned with cranial to the left. a: thoracic duct; b: left tracheal trunk; c: left superficial cervical artery; e: left external jugular vein; g: left axillary artery; h: left internal thoracic artery; i: left common carotid artery; j: bicarotid trunk; k: left subclavian artery; l: aorta; m: heart; n: thymus; o: oesophagus; p: left costocervical artery; q: left costocervical vein; r: the vertebral and sternal cutting edges of the first left rib; g: left costocervical vein; h: left internal thoracic artery; s: left vagosympathetic trunk t: left vagus nerve u: left internal thoracic vein; v: left cranial lobe of the lung; w: brachiocephalic trunk; x: cranial vena cava; dotted line: left phrenic nerve

We found that the anatomic positions of the thoracic duct and the left tracheal trunk where they joined the cardiovascular system were quite similar in all 15 of the Large White piglets in this study. In 12 of the 15 piglets (80 %), the thoracic duct and the left tracheal trunk joined the left external jugular vein craniomedial to the first rib on the left side. In three pigs, the ampulla of the thoracic duct was located close to the junction of the left external jugular vein and the left subclavian vein, more medial than craniomedial to the first rib on the left side. The left tracheal trunk was single at its proximal end cranial to its point of entry into the external jugular vein in all 15 Large White piglets. We did not find additional branches of the left tracheal trunk at its segment proximal to its point of entry into the external jugular veins in any pig. However, in one pig, the left tracheal trunk anastomosed with the thoracic duct (the ampulla of the thoracic duct) at the craniomedial thoracic inlet (Fig. 1).

**Fig. 2:**
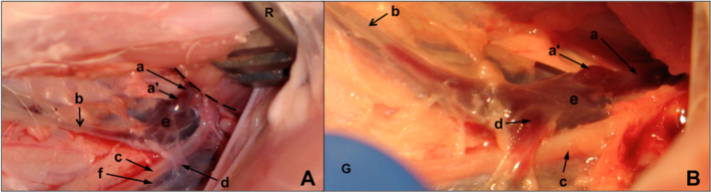
Anatomic position of the thoracic duct and its ampulla at the cranial thoracic inlet on the left. The figures show the region cranial to the thoracic inlet on the left in a 7-week old Large White pig. The pictures are positioned with cranial to the left. **A**: The thoracic duct (a), emerging from the thoracic cavity, and its ampulla (a’) that joins the external jugular vein craniomedially to the first rib (the dashed line marks the cranial border of the first rib) on the left could be identified. **B**: The dorsal craniolateral aspect of the left external jugular vein (e) at its junctions with the thoracic duct (a), left tracheal trunk (b), and the efferent lymphatic (d) of the left dorsal superficial cervical lymph nodes. c: left superficial cervical artery; f: left superficial cervical vein. (R: retractors; G: gloves)

In Fig. 2A, a short segment of the thoracic duct can be seen emerging from the thoracic cavity, and the junction of its ampulla with the left external jugular vein can be observed, located craniomedial to the first left rib. The ampullae of the left tracheal trunk and the efferent lymphatic of the left dorsal superficial cervical lymph node(s), which are located cranial to the ampulla of the thoracic duct, also inserted into the left external jugular vein (Fig. 2B). The magenta-colour of the thoracic duct was probably due to the presence of abundant red blood cells (RBCs) and their derivatives in the lymph since the animal had previously had severe abdominal haemorrhage due to sample collections associated with another study. In Fig. 2, clear and reddish lymph with an obvious boundary can be observed in the left tracheal trunk (b) proximal to the left external jugular vein (e). It is likely that there are valves located at the demarcation between the lymph with different colours.

Fig. 1 shows the course of the thoracic duct (indicated by ➞) and the tracheal trunk (labelled with →) in the cervicothoracic region on the left. Other major structures in this region are also identified and the relationship of the thoracic duct to these structures is clearly depicted. While an anastomosis of the left tracheal trunk and the thoracic duct was found in this animal, the ampulla of the thoracic duct was, as found in most of the specimens, located craniomedial to the first left rib.

**Fig. 3:**
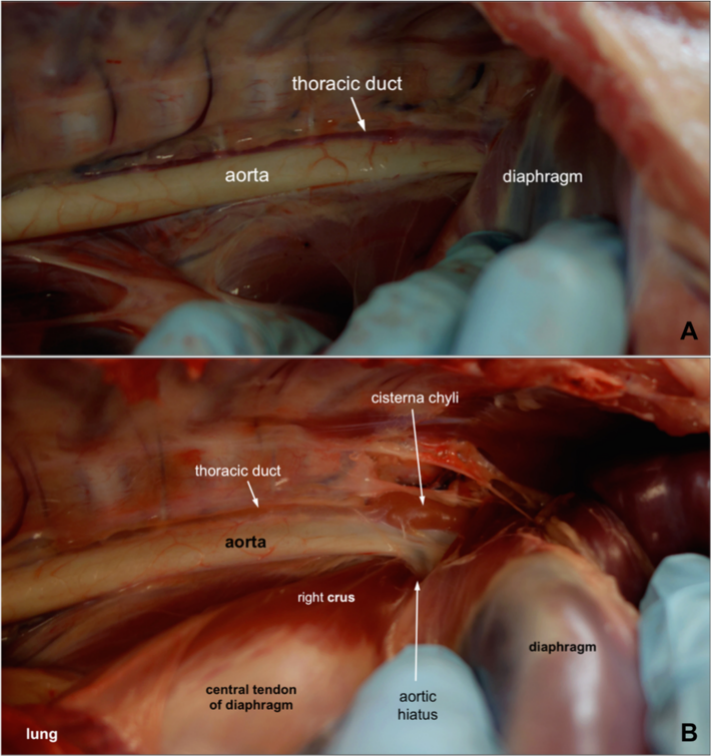
The course of the thoracic duct and it junction with the cisterna chyli in the thoraco-abdominal junction on the left. Dissections were performed on a 7-week old Large White pig immediately after it was euthanised to expose the lateral aspect of the left thoraco-abdominal junction. The pictures are arranged with cranial to the left. **A**: The dorsal parts of the left lung were retracted ventrally to expose the thoracic duct that runs along the dorsal border of the aorta. The thoracic duct is red tinged probably due to the presence of large numbers of RBCs. **B**: The diaphragm has been incised lateral and dorsal to the aortic hiatus and the left crus of the diaphragm retracted caudally to expose the cisterna chyli and its junction with the caudal end of the thoracic duct in the cranial sub-lumbar region of the abdominal cavity. The reddish colour of the thoracic duct became paler due to influxes of more body fluid

While Figs. 1 and 2 show the colour of the thoracic ducts of pigs that underwent prior abdominal tissue collections, Fig. 3 shows the appearance of the thoracic duct of a normal pig dissected immediately after euthanasia. The red appearance of the thoracic ducts was a finding in all four pigs that were dissected immediately after euthanasia. No dissections on the bodies caudal to the diaphragm were performed on these pigs before approaching the thoracic ducts. In Fig. 3A, the left thoracic wall has been removed and the dorsal parts of the left lung retracted ventrally to expose a left lateral view of the thoracic duct running along the dorsal border of the aorta. In Fig. 3B, the diaphragm has been incised lateral and dorsal to the aortic hiatus and the left crus of the diaphragm retracted caudally to expose the cisterna chyli and its junction with the caudal end of the thoracic duct in the cranial sub-lumbar region of the abdominal cavity. The reddish colour of the thoracic duct became paler at about 40 min post-euthanasia as a result of more body fluid draining into the thoracic duct.

By identifying the cisterna chyli and its junction with the thoracic duct and tracking the course of the thoracic duct downstream to the cervicothoracic region, we also confirmed the course and position of the thoracic duct in the cranial thorax in these animals.

## Discussion

In this paper we present photographs showing the course of the thoracic duct, the left tracheal trunk, and the efferent lymphatic of the left dorsal superficial cervical lymph node(s) at their points of entry to the left external jugular vein in fresh porcine specimens. These images also show the relationships between the lymphatic vessels and other anatomical structures in the cervicothoracic region on the left side in pigs. Findings in our study expand the understanding of the locations of the major lymphatic vessels in the left cervicothoracic region of pigs.

From our experience of studying lymphatic anatomy in sheep, dogs, and pigs, variations in the course and number of lymphatic vessels in different animals is not uncommon. Indeed, several references also identified the anatomical variations in lymphatic vessels [3, 5, 6]. The results of this study however, found that there was a single left tracheal trunk at its proximal end and point of entry in all 15 individual Large White pigs. These results therefore suggest that the anatomy of the left tracheal trunk at its proximal end where it enters the external jugular may be reasonably consistent in Large White pigs. It is possible however, that the sample number of pigs in the current study was not sufficiently high to observe uncommon variations. Results by Gomercic et al. for example, showed that Yorkshire pigs may sometimes have double tracheal trunks rather than the more commonly found single trunk [3].

As shown in Fig. 3, the colours of the thoracic ducts were found to be grossly reddish instead of translucent light yellowish in all four pigs that were euthanised specifically for this thoracic duct anatomy study. These results are compatible with previous observations [7, 8]. Nevertheless, the presence of large numbers of RBCs was not found in the tracheal trunks and the efferent lymphatic vessels of the dorsal superficial cervical lymph centres. The colours of these two lymphatic vessels were translucent and colourless to very light straw-coloured. Binns et al. also observed that the intestinal lymph of pigs was milky and not visibly bloody [7]. They speculated that the presence of large numbers of RBCs in the thoracic duct might result from the anastomoses of lymphatics and veins. The cause of these observed variations in RBC numbers in different lymphatics needs further investigation. Cannulation of individual lymphatic ducts for collection of lymph for measurement of blood cell numbers at different lymphatic sites could be a useful initial step.

## Conclusions

**Fig. 4:**
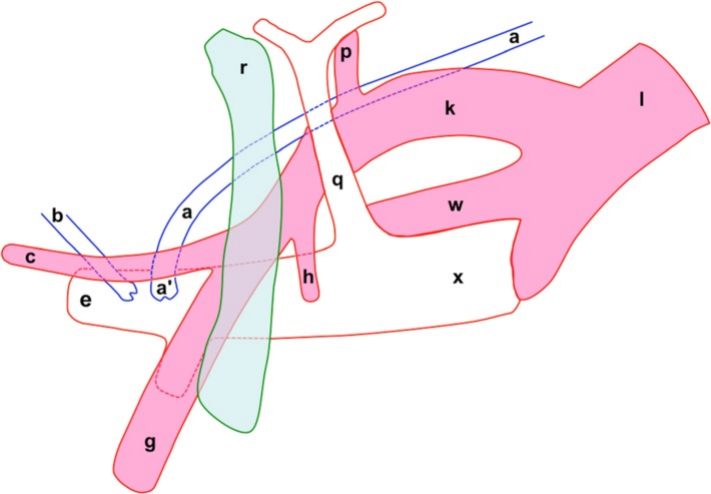
Structural relationships of the thoracic duct and its entry into the external jugular vein cranial to the first rib on the left side. The point of entry (a’) of the thoracic duct into the external jugular vein was located craniomedial to the first rib (r) on the left. The drawing is prepared with cranial to the left. a: thoracic duct; b: left tracheal trunk; c: left superficial cervical artery; e: left external jugular vein; g: left axillary artery; h: left internal thoracic artery; k: left subclavian artery; l: aorta; p: left costocervical artery; q: left costocervical vein; r: first left rib; w: brachiocephalic trunk; x: cranial vena cava

The results of this study suggest that it is feasible to access the thoracic duct cranial to the thoracic inlet with minor tissue damage and without thoracotomy in Large White piglets. As shown in Fig. 2A, the junction of the thoracic duct and the external jugular vein was located craniomedial to the first rib on the left in 80 % of the 4 to 7-week-old Large White piglets. In this image, it is obvious that the connective tissue medial to the first rib is undamaged. This finding suggests that the thoracic duct can indeed be accessed without any surgical incision into the chest wall. With this high consistency in the thoracic duct location at the cranial thoracic inlet, the chances for approaching the thoracic duct successfully become more promising. A drawing annotating the positional relationships between the point of entry of the thoracic duct into the external jugular vein and the first rib and several nearby major blood vessels in the cervico-thoracic region on the left is presented in Fig. 4.

Based on these findings, we will develop a cannulation model, enabling both the thoracic duct and the tracheal trunk (left) to be catheterised without thoracotomy, for long-term efferent pulmonary and nasal lymph collection using Large White piglets. Long-term catheterisation of the thoracic duct in pigs allowing *in vivo*, *in situ*, and real-time lymph collection will be useful in biomedical research in the fields of pulmonology, gastro-enterology, pathobiology, pharmacokinetics and immunology studies [9]. Obtaining lymph supernatants of the draining tissues provides an approach to study the presence of, or changes in, molecules like exosomes, drugs and cytokines in studies such as those on the pharmacokinetics of medicines.

## Methods

The cadavers of 15 Large White piglets about 4–7 weeks old were used for this study. The cadavers of 11 of the piglets were collected after they had been euthanised at the end of animal trials not associated with this study –that were conducted at the Faculty of Veterinary and Agricultural Science (FVAS) animal facility. Collections of multiple tissues in the abdominal cavities had been performed in these 11 pigs just before they were euthanised. Consequently, the presence of abundant RBCs in the thoracic duct was predicted in these specimens.

In addition to the 11 pig cadavers, four Large White piglets (aged 4–7 weeks) were housed in pens within the FVAS animal facility. These pigs were fed with commercial pellets and allowed access to water ad libitum. -They were euthanised by intravenous injection of Lethabarb (Pentobarbitone Na 325 mg/ml) into the right external jugular vein and dissected immediately following euthanasia. The University of Melbourne Animal Experimentation Ethics Committee approved all experimental procedures.

The dissection procedures were designed based on swine anatomical information from anatomical textbooks [10, 11] and modified from the surgical procedures in the previous study in sheep [2]. Dissections for approaching the thoracic duct were made through the caudal cervical region, craniomedial and slightly dorsal to the left shoulder joint. After the initial skin incision, and where possible, blunt dissection was mostly applied to avoid damaging the major blood vessels, lymphatics [11], and nerves in this region after skin incision. To generate enough space for deep dissections, blood vessel branches of the left superficial cervical artery and vein that coursed caudo-laterally to the suprascapular regions were ligated and transected. However, ligation and transection of other main blood vessels was generally not performed, in order to retain their relationships to the lymphatic vessels.

In most preparations, the left tracheal trunk, the efferent lymphatic of the dorsal superficial cervical lymph node(s), and the external jugular vein at the caudal cervical region were initially identified. The two lymphatic vessels were then followed downstream to the points of entry (ampullae) where they joined with the left external jugular vein. The thoracic duct - a large lymphatic vessel close to the first left rib that exits the thoracic cavity via the thoracic inlet - and its ampulla, which is beside the ampulla of the left tracheal trunk, were carefully isolated from the peripheral tissue. To confirm the identification of the thoracic duct and its relationship to the other anatomical structures adjacent to the thoracic inlet, the left chest wall was carefully removed to expose the thoracic duct in the thoracic cavity. We also dissected out part of the diaphragm to expose the junction between the thoracic duct and the cisterna chyli.

## Abbreviations

FVAS: The faculty of veterinary and agricultural science
RBCs: Red blood cells

## References

[1] Kassai T, Shnain A, Kadhim J, Altaif K, Jabbir M (1972). Collection of lymph from sheep by the cannulation of the thoracic duct and an application of the method in hosts infected with Dictyocaulus filaria. Magyar Allatorvosok Lapja.

[2] Yen HH, Wee JL, Snibson KJ, Scheerlinck JP (2009). Thoracic duct cannulation without thoracotomy in sheep: a method for accessing efferent lymph from the lung. Vet Immunol Immunopathol.

[3] Gomercic MD, Vukicevic TT, Gomercic T, Galov A, Fruk T, Gomercic H (2010). The cisterna chyli and thoracic duct in pigs (Sus scrofa domestica). Vet Med.

[4] Saar LI, Getty R, Getty R (1975). Porcine lymphatic system. Sisson and Grossman’s The Anatomy of the Domestic Animals.

[5] Landolt CC, Matthay MA, Staub NC (1981). Anatomic variations of efferent duct from caudal mediastinal lymph node in sheep. J Appl Physiol.

[6] Lascelles AK, Morris B (1961). Surgical techniques for the collection of lymph from unanaesthetized sheep. Q J Exp Physiol Cogn Med Sci.

[7] Binns RM, Hall JG (1966). The Paucity of lymphocytes in the Lymph of Unanaesthetised Pigs. Br J Exp Pathol.

[8] Ashitate Y, Tanaka E, Stockdale A, Choi HS, Frangioni JV (2011). Near-infrared fluorescence imaging of thoracic duct anatomy and function in open surgery and video-assisted thoracic surgery. J Thorac Cardiovasc Surg.

[9] Chanoit G, Ferre PJ, Lefebvre HP (2007). Chronic lymphatico-venous bypass: surgical technique and aftercare in a porcine model. Interact Cardiovasc Thorac Surg.

[10] Nickel R, Schummer A, Seiferle E (1981). The circulatory system, the skin, and the cutaneous organs of the domestic mammals / by August Schummer, Helmut Wilkens, Bernd Vollmerhaus, and Karl-Heinz Habermehl; translation by Walter G. Siller and Peter A. L. Wright.

[11] Saar LI, Getty R, Getty R (1975). Porcine lymphatic system. Sisson and Grossman’s The Anatomy of the Domestic Animals.

